# The mediating role of sleep quality on the relationship between physical activity and social anxiety disorder among Chinese college freshmen

**DOI:** 10.3389/fpsyg.2025.1599041

**Published:** 2025-07-15

**Authors:** Yingying Liu, Zizheng Nie, Haokai Dong, Linpei Shou, Jiaoyang Xu, Qinglong Cao, Yitian Shi, Chenyang Wu, Mu Zhang, Jun Yang, Shufen Han

**Affiliations:** ^1^Department of Nutritional and Toxicological Science, School of Public Health and Nursing, Hangzhou Normal University, Hangzhou, Zhejiang, China; ^2^School of Public Health and Nursing, Hangzhou Normal University, Hangzhou, Zhejiang, China

**Keywords:** physical activity, social anxiety disorder, sleep quality, mediation analysis, college freshmen

## Abstract

**Objectives:**

Social anxiety disorder (SAD) profoundly impacts the wellbeing of college students, both physically and psychologically. This study aimed to examine the mediating role of sleep quality on the relationship between physical activity and SAD among Chinese college freshmen.

**Methods:**

This was a cross-sectional study enrolling 1,408 participants through convenience sampling. Participants self-reported their physical activity, sleep quality, and social anxiety status using an electronic questionnaire.

**Results:**

The overall prevalence of SAD was 33.05%. A significant negative association was observed between physical activity and SAD (adjusted β = −0.054, 95% CI: −0.053 to −0.001). Mediation analysis indicated that sleep quality may mediated 24.33% of the association between physical activity and SAD (indirect effect estimate = −0.009, direct effect estimate = −0.028, *p* < 0.01).

**Conclusions:**

Less physical activity is associated with SAD among Chinese college freshmen, and this relationship is partially mediated by poor sleep quality. It is recommended that educational institutions implement programs to promote regular physical exercise and enhance sleep quality to mitigate the impacts of SAD in this population.

## 1 Introduction

The college years represent a crucial period in the development of students, marking their transition from late adolescence to the early stages of adulthood (Arnett, 2000). The first year of college is an important turning point, fraught with various potential stressors for incoming freshmen as they acclimate to their newfound responsibilities and independence. Given these substantial life changes that occur during this period, it is not surprising that anxiety is common experience among first-time college students. Anxiety is widely acknowledged as the most common mental health problem among college students worldwide, with an estimated prevalence rate of 41.6% (Bayram and Bilgel, 2008; Mao et al., 2019). Anxiety not only affects their academic performance but also their physical and mental health (Bruffaerts et al., 2018; BlackDeer et al., 2023), and even increases dropout rates (Kivlighan et al., 2021). Social anxiety disorder (SAD), also known as social phobia, stands out among anxiety disorder due to its core feature of interpersonal dysfunction. It is characterized by a pronounced and persistent fear of one or more social situations, such as interactions with strangers or peers, attending parties, or delivering speeches (American Psychiatric Association, 2013). SAD is a prevalent and incapacitating psychiatric disorder, with an estimated lifetime prevalence rate of ~12% (Kessler et al., 2005).

There is a growing body of evidence suggesting that physical activity is widely acknowledged as an effective strategy for alleviating anxiety and improving the physical and mental wellbeing of students (Stubbs et al., 2017; McDowell et al., 2019; Rodríguez-Romo et al., 2022). Encouraging physical activity has been identified as a promising preventive measure for anxiety and other mental disorders, and it has the potential to be a cost-effective and practical way for reducing the prevalence of anxiety and anxiety-related disorders (Kandola et al., 2018). However, most college students do not meet the recommended activity levels outlined in the physical activity guidelines, which advocate for at least 150 minutes of moderate-intensity aerobic exercise or 75 min of high-intensity aerobic exercise per week (Irwin, 2004; Keating et al., 2005; Barr-Anderson et al., 2018). This shortfall is detrimental to their health, particularly with regard to mitigating social anxiety.

Sleep-related problems are highly prevalent among college students, affecting ~10% to 50% of this population (Dinis and Bragança, 2018). A recent review highlights the beneficial effects of engaging in moderate to intense physical activity on improving sleep quality (Sejbuk et al., 2022). Improved sleep quality, in turn, may contribute to significant advancements in students' psychobiological health, particularly in alleviating anxiety disorders (Scott et al., 2021; Saravanan et al., 2024). Studies have shown that poor sleep quality was associated with a higher prevalence of anxiety among college students (Milojevich and Lukowski, 2016; Ghrouz et al., 2019). Furthermore, inadequate sleep may lead to increased social avoidance and hinder extinction learning, thereby exacerbating the risk of SAD (Ben Simon and Walker, 2018).

The relationship between physical activity, sleep quality, and SAD can be understood through the established biopsychosocial model. From a biological standpoint, physical exercise has been demonstrated to modulate neurotransmitters such as dopamine, endorphins, and serotonin (Ratey, 2008), with serotonin dysfunction being linked to the development of sleep disorders (Ursin, 2002). Self-determination theory posits that physical activity can alleviate social anxiety and promote social participation by satisfying the psychological needs for autonomy, competence, and relatedness (Deci and Ryan, 1985), while high-quality sleep can reduce emotional reactivity to stress by facilitating the consolidation of emotional memories (Walker and van der Helm, 2009). However, the role of sleep quality on the relationship between physical activity and SAD among the college freshmen has not been extensively studied. Therefore, we propose that sleep quality acts as a partial mediating role between physical activity and SAD. To investigate this hypothesis, we conducted this cross-sectional study to evaluate the mediating role of sleep quality on the relationship between physical activity and SAD within this population.

## 2 Methods

### 2.1 Study design and participants

A cross-sectional study was performed and related information was collected from first-year students at Hangzhou Normal University between November and December 2023. To ensure a manageable and representative sample for the study, a convenience cluster sampling method was used to select the entire class of students from different departments of Hangzhou Normal University. The inclusion criteria included male or female first-year students between 18 and 20 years, who were capable of following the study instructions and conscientiously completing the survey questionnaire on the WJX platform (www.wjx.cn). The online survey was conducted in compliance with national and international regulations, adhering to the principles outlined in the Declaration of Helsinki. We meticulously explained the study purpose to the class presidents, and provided standardized uniform training to all investigators, with the aim of ensuring the accuracy and dependability of the data collected. Subsequently, each investigator entered the freshmen's classrooms, where they distributed questionnaire through an online platform and provided face-to-face professional guidance during the questionnaire completion process. Students who self-reported any physical activity limitations or contraindications were excluded from the study. Additionally, those with medical diagnostic evidence confirming a diagnosis of sleep apnoea syndrome or primary insomnia were also excluded. The research procedure was approved by the Ethics Committee of Hangzhou Normal University (No. 202300012). Each participant was asked to indicate his or her willingness in this study and signed an online informed consent. All participants were informed that their personal information would be kept confidential, and their privacy rights would be protected.

### 2.2 Sample size determination

The sample size for the cross-sectional study was determined using the following formula: (Cochran, 1977) *p* represents the population rate and q is equal to 1-p; d stands for the allowable error, and $Z_{1-\alpha /2}^{2}$ indicates the statistic for the significance test. The *p* value was established at 0.079, based on the reported prevalence of SAD (Izgiç et al., 2004). And the d value was set at 0.2 × *p*, α at 0.05, and $Z_{1-\alpha /2}^{2}\;$at 1.96 for this study. By substitute these values into the following formula, we calculated the initial sample size. After considering a 20% increase to the initial sample size, the final calculated sample size for this study is 1,344 participants.


$$\begin{array}{l} n=\frac{Z_{1-\alpha /2}^{2}\times pq}{d^{2}} \end{array}$$

Initially, a total of 1,458 first-year students were invited to participate in an online survey. After screening for inadequate response time with < 180 s, incomplete questionnaires, obvious logical errors, and missing school number, the final sample size for the study comprised 1,408 subjects, with a valid response rate of 96.50%.

### 2.3 Physical activity assessment

The Physical Activity Rating Scale (PARS)-3 is a self-report questionnaire designed to assess the level of routine physical activity in individuals. The PARS-3 scale is utilized to measure the intensity, duration, and frequency of each activity and has been applied to Chinese college students, with a test-retest reliability of 0.82 (Liang, 1994). The total score for physical activity was calculated according to the following formula: exercise volume = physical activity intensity × duration × frequency. The PARS-3 evaluates the intensity and frequency on a 5-point scale, with each level rated from 1 to 5. The duration is assessed on a scale from 0 to 4, with 4 indicating the greatest amount of time spent on the activity and 0 indicating the least. Each level is assigned a score ranging from 0 and 5, which corresponds to the individual's self-reported level of activity. The scores for intensity, duration and frequency are combined to yield a total score, with the maximum possible score being 100 points. The exercise rating criteria for PARS-3 are classified into three distinct levels: less than 19 points for low volume, 20 to 42 points for medium volume, and 43 points or above for high volume (Liang, 1994). The Cronbach's α coefficient for PARS-3 scale in this study was 0.878.

### 2.4 Definition of SAD

The Interaction Anxiousness Scale (IAS) is a measurement tool developed by Leary (1983), which is used to assess the propensity for experiencing subjective social anxiety. especially in face-to-face interaction scenarios that demand immediate feedback. The scale consists of 15 self-report items, including 11 positively worded and 4 negatively worded statements, each describing a potential anxiety-inducing scenario in social interactions. Respondents were asked to assess the relevance of each statement to their own experiences using a five-point Likert scale, with rating from 1 to 5, indicating “not at all like me” to “very much like me,” respectively. Total scores ranged from 15 to 75, with higher total IAS scores indicating more severe social anxiety. According to the literature reported (Cao et al., 2016), an IAS score of 50 or above was predictive of the presence of SAD, while a score below 50 was considered indicative of the absence of SAD. The Cronbach's α coefficient for IAS scale in this study was 0.891.

### 2.5 Sleep quality assessment

Sleep quality was assessed using the Pittsburgh Sleep Quality Index (PSQI) developed by Buysse et al. (1989). The PSQI is a widely used scale for evaluating subjective sleep disturbance in both clinical and research settings. The scale consists of 18 items scored on seven aspects of sleep, including subjective sleep quality, sleep latency, sleep duration, habitual sleep efficiency, sleep disturbance, used sleep medication and daytime dysfunction in the past month. Each aspect is scored from 0 (indicating not in the past month or very good) to 3 (indicating three or more times 1 week or very bad). The total score for the PSQI scale, which ranges from 0 to 21, is the sum of the scores for its seven aspects of sleep, with higher scores indicating poorer sleep quality. A score of <8 indicates no sleep disorder, while scores of 8 or above suggest the presence of sleep disorder (Lund et al., 2010). The Cronbach's α coefficient for PSQI scale in this study was 0.627.

### 2.6 Covariates

Socio-demographic information of the students, including age, gender, nationality, regional origin with the east, area of residence, status as an only child, major, repeat student status, monthly living expenses, and involvement in student clubs, was collected using a standardized structured questionnaire. Nationality was classified as Han Chinese or other minorities. Based on the economic conditions and dietary habits, the regional origin with the east primarily included Guangdong Province, Zhejiang Province, Fujian Province, Jiangsu Province and Shanghai City. Area of residence was categorized as rural or urban. According to the professional classification established by the Ministry of Education of China, majors were classified as literature, science/management, engineering/agriculture, and medicine. Monthly living expenses were grouped as less than 1200, 1200–1999, 2000–2999 and more than 3000 *yuan*. Other covariates were represented by dichotomous variables, coded as either “yes” or “no.”

### 2.7 Statistical analysis

A chi-square test and a student's *t* test were conducted to assess whether students with SAD and students without SAD differed on socio-demographic characteristics. Linear correlation analysis was used to examine the relationships between physical activity, SAD, and sleep quality. Multiple linear regression models were used to analyze the relationship between physical activity and SAD: in model 1, all pertinent variables from prior covariates were adjusted, including age, gender, nationality, regional origin with the east, area of residence, status as an only child, major, repeat student status, monthly living expenses, and involvement in student clubs; model 2 was further adjusted for sleep quality based on model 1. Additionally, Cohen's d effect sizes were calculated for the different exercise intensity groups, including low, medium and high (Cohen, 2013).

To examine whether sleep quality mediated the relationship between physical activity and SAD, we conducted a mediation analysis using the SPSS PROCESS macro, version 4.1 (model 4), developed by Hayes (2012). Mediation hypotheses regarding the role of sleep quality on the relationship between physical activity and SAD were tested using the bias-corrected bootstrap method, which involved 1,408 samples to calculate 95% confidence intervals (CIs). All covariates, including age, gender, nationality, regional origin with the east, area of residence, status as an only child, household registration, major, repeat student status, monthly living expenses, and involvement in student clubs, were adjusted during the mediating effect analysis. Mediation analysis was performed using SPSS software (version 26.0; IBM Corp., Armonk, NY, USA), while all other statistical analyses were conducted using R software (version 4.4.3; R Development Core Team, Vienna, Austria).

## 3 Results

### 3.1 Sample characteristics

The socio-demographic characteristics of the participants are summarized in Table 1. A total of 1,408 college freshmen, including 531 males and 877 females with an average age of 18.5 years (SD = 0.79), were enrolled in the present study. Based on the IAS evaluation, 464 students were identified as having SAD, with a significantly higher prevalence in females than in males (36.3 vs. 27.5%, *p* < 0.001). Additionally, a total of 805 students reported low levels of physical activity, with a mean PARS-3 score of 8.86 (SD = 5.64); 378 students self-reported experiencing sleep disorder, with a prevalence rate of 26.8%.

**Table 1:** Socio-demographic characteristics of college freshmen based on their SAD status.

**Variables, *n* (%)**	**With SAD (*n* = 464)**	**Without SAD (*n* = 944)**	**Total sample (*n* = 1,408)**	***p* value**
Age, M (SD)^*^	18.45 (0.71)	18.52 (0.82)	18.5 (0.78)	0.078
**Gender**				<0.001
Male	146 (31.5)	385 (40.8)	531 (37.7)	
Female	318 (68.5)	559 (59.2)	877 (62.3)	
**Nationality**				0.059
Han Chinese	436 (94.0)	858 (90.9)	1,294 (91.9)	
Other minorities	28 (6.0)	86 (9.1)	114 (8.1)	
**Regional origin with the east**				0.083
Yes	334 (72.0)	635 (67.3)	969 (68.8)	
No	130 (28.0)	309 (32.7)	439 (31.2)	
**Area of residence**				0.172
Rural	280 (60.3)	532 (56.4)	812 (57.7)	
Urban	184 (39.7)	412 (43.6)	596 (42.3)	
**Status as an only child**				0.045
Yes	158 (34.1)	375 (39.7)	533 (37.9)	
No	306 (65.9)	569 (60.3)	875 (62.1)	
**Major**				0.844
Literature	137 (29.5)	266 (28.2)	403 (28.6)	
Science/Management	114 (24.6)	244 (25.8)	358 (25.4)	
Engineering/Agriculture	40 (8.6)	91 (9.6)	131 (9.4)	
Medicine	173 (37.3)	343 (36.4)	516 (36.6)	
**Repeat student status**				0.817
Yes	48 (10.3)	103 (10.9)	151 (10.7)	
No	416 (89.7)	841 (89.1)	1,257 (89.3)	
**Monthly living expenses**				0.101
<1200 *yuan*	34 (7.3)	74 (7.8)	108 (7.7)	
1200–1999 *yuan*	202 (43.5)	390 (41.3)	592 (42.0)	
2000–2999 *yuan*	203 (43.8)	394 (41.7)	597 (42.4)	
≥3000 *yuan*	25 (5.4)	86 (9.2)	111 (7.9)	
**Involvement in student clubs**				0.109
Yes	299 (64.4)	650 (68.9)	949 (67.4)	
No	165 (35.6)	294 (31.1)	459 (32.6)	
**Physical activity**				
PARS-3 scores, M (SD)^*^	20.84 (18.56)	22.91 (20.21)	22.23 (19.70)	0.057
**Exercise intensity rating**				0.026
Low exercise	288 (62.1)	517 (54.8)	805 (57.1)	
Medium exercise	108 (23.2)	276 (29.2)	384 (27.3)	
High exercise	68 (14.7)	151 (16.0)	219 (15.6)	
**Sleep characteristics**				
PSQI scores, M (SD)^*^	6.94 (3.01)	5.46 (2.93)	5.80 (3.00)	<0.001
**Sleep disorder**				
Yes	165 (35.6)	213 (22.6)	378 (26.8)	<0.001
No	299 (64.4)	731 (77.4)	1,030 (73.2)	

* Age, PARS-3 scores and PSQI scores are continuous measures; all other variables are categorical. SAD, social anxiety disorder; PARS-3, physical activity rating scale-3; PSQI, pittsburgh sleep quality index.

First-year students who were identified as having low exercise volume based on the PARS-3 assessment or who experienced sleep disorder based on PSQI scale, were found to be more likely to suffer from SAD. Students with SAD had significantly higher mean PSQI scores (M = 6.94, SD = 3.01) compared to those without SAD (M = 5.46, SD = 2.93). Meanwhile, the mean PARS-3 scores for students with SAD (M = 20.84, SD = 18.56) were slightly lower than those without SAD (M = 22.91, SD = 20.21), but the difference was not statistically significant. Significant differences were also observed between the two groups, those with SAD and those without, concerning gender and status as an only child were also observed between SAD status (all *p* < 0.05). The Cohen's d for the difference in IAS scores between the high-intensity and moderate-intensity exercise groups was −0.051 (95% CI, −0.217 to 0.116, *p* = 0.055). In contrast, the Cohen's d for the difference between the high-intensity and low-intensity exercise groups, as well as between the moderate-intensity and low-intensity groups, were −0.281 (95% CI, −0.431 to −0.131, *p* < 0.001) and −0.238 (95% CI, −0.360 to −0.116, *p* < 0.001), respectively.

### 3.2 Correlation and regression analysis

**Figure 1:**
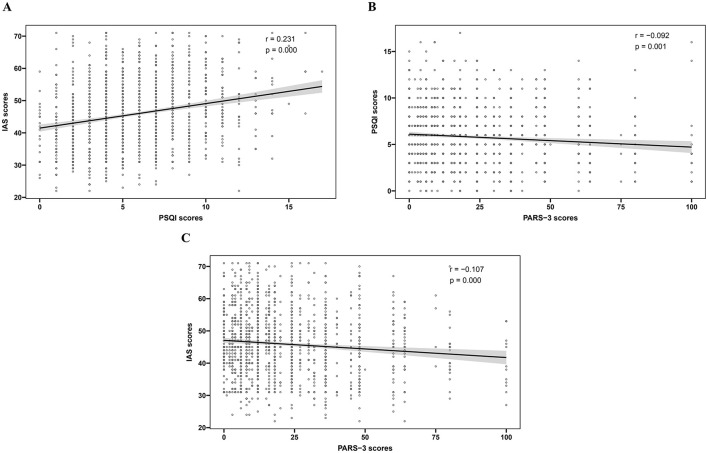
Scatter plots of correlation of PARS-3 scores, IAS scores, and PSQI scores. **(A)** The correlation between PSQI scores and IAS scores; **(B)** The correlation between PARS-3 scores and PSQI scores; **(C)** The correlation between PARS-3 scores and IAS scores.

Figure 1 shows that PARS-3 scores were negatively correlated with IAS scores (*r* = −0.092, *p* = 0.001) and PSQI scores (*r* = −0.107, *p* < 0.001). PSQI scores was significantly associated with SAD (*r* = 0.231, *p* < 0.001). The results of multiple linear regression analysis evaluating the relationship between physical activity and SAD, are presented in Table 2. After adjusting for covariates including age, gender, nationality, regional origin with the east, area of residence, status as an only child, household registration, major, repeat student status, monthly living expenses, and involvement in student clubs, model 1 showed a statistically significant association between PARS-3 scores and IAS scores (β = −0.071, 95% CI, −0.062 to −0.009, *p* = 0.010). Upon further adjustment for the PSQI scores, the association between PARS-3 scores and ISA scores remained significant (β = −0.054, 95% CI, −0.053 to −0.001, *p* = 0.042), indicating a partial mediating role of sleep quality on the relationship. It was noteworthy that this association was particularly significant among female subjects (Gender: Female, β = 2.097, *p* < 0.001), underscoring the importance of gender.

**Table 2:** Regression analysis between physical activity and social anxiety disorder in college freshmen.

**Outcome variables**	**Model 1**				**Model 2**			
	β	***p***	**95% CI**		β	***p***	**95% CI**	
			**Lower**	**Upper**			**Lower**	**Upper**
PARS−3(scores)	−0.071	0.010	−0.062	−0.009	−0.054	0.042	−0.053	−0.001
Gender: Female	2.336	0.000	1.194	3.479	2.097	0.000	0.985	3.208
Age	−0.619	0.066	−1.280	0.041	−0.877	0.008	−1.522	−0.233
Nationality: Others	−1.977	0.053	−3.977	0.023	−2.128	0.032	−4.073	−0.183
Regional origin with the east: No	−0.738	0.244	−1.980	0.504	−0.862	0.161	−2.070	0.345
Area of residence: Urban	−0.133	0.811	−1.220	0.954	−0.232	0.667	−1.289	0.825
Status as an only child: No	1.280	0.024	0.172	2.388	1.140	0.038	0.062	2.218
**Major**								
Science/Management	0.480	0.508	−0.943	1.903	0.584	0.408	−0.800	1.968
Engineering/Agriculture	0.375	0.715	−1.637	2.387	0.557	0.576	−1.399	2.514
Medicine	0.447	0.504	−0.863	1.757	0.915	0.160	−0.363	2.193
Repeat student status: Yes	−0.951	0.258	−2.601	0.698	−0.994	0.224	−2.598	0.610
Involvement in student clubs: No	2.234	0.000	1.091	3.378	2.412	0.000	1.299	3.524
**Monthly living expenses**								
1200–1999 *yuan*	0.413	0.686	−1.587	2.412	−0.035	0.972	−1.981	1.912
2000–2999 *yuan*	−0.676	0.521	−2.744	1.391	−1.000	0.329	−3.012	1.011
≥ 3000 *yuan*	−3.109	0.022	−5.771	−0.447	−3.665	0.006	−6.256	−1.074
PSQI (scores)	–	–	–	–	0.234	0.000	0.600	0.934
Constant	55.730	0.000	43.044	68.416	56.303	0.000	43.968	68.638
Observations	1,408				1,408			
*R* ^2^	0.049				0.038			
Adjusted *R*^2^	0.101				0.091			
Residual standard error	9.643 (*df* = 1407)				9.376 (*df* = 1406)			
F Statistic (df; *p* value)	4.753 [*df* = (15,1407); *p* value < 0.001]				9.807 [*df* = (16,1406);*p* value < 0.001]			

Model 1: multiple linear regression analysis between PARS–3 scores and ISA scores.

Model 2: multiple linear regression analysis between PARS–3 scores and ISA scores adjusted by PSQI scores. Models were adjusted for all covariates, including age, gender, nationality, regional origin with the east, area of residence, status as an only child, household registration, major, repeat student status, monthly living expenses, and involvement in student clubs.

### 3.3 Mediation analysis

**Figure 2:**
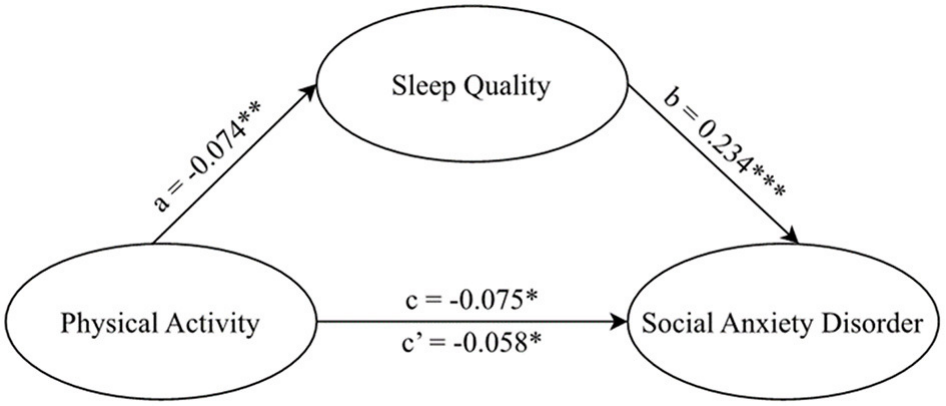
Model of mediating effect of sleep quality on the relationship between physical activity and social anxiety disorder among college freshmen.

As demonstrated in Table 3 and Figure 2, after adjusting for all the aforementioned covariates, the results indicated that sleep quality might partially mediate the relationship between physical activity and SAD (Effect = −0.009, SE = 0.004, 95% CI, −0.016 to −0.002). The mediating effect was found to account for 24.33% of the total effect (Effect = −0.037, SE = 0.014, 95% CI, −0.064 to −0.011).

**Table 3:** The mediating effect of sleep quality on the association between physical activity and social anxiety disorder.

**Effect**	**Effect value**	**Boot SE**	**Boot CI lower**	**Boot CI upper**	**Relative mediation effect (%)**
Total effect	−0.037	0.014	−0.064	−0.011	100.00
Direct effect	−0.028	0.013	−0.055	−0.003	75.67
Indirect effect	−0.009	0.004	−0.016	−0.002	24.33

Model was adjusted for all covariates, including age, gender, nationality, regional origin with the east, area of residence, status as an only child, household registration, major, repeat student status, monthly living expenses, and involvement in student clubs.

## 4 Discussion

For first-year college students, changes in their environment can be a trigger for alterations in their mental and physical wellbeing (Li et al., 2023). It was estimated that approximately one-third of college freshmen will encounter a variety of psychological challenges, including the distress of associated with SAD (Bruffaerts et al., 2018; Li and Sun, 2023). The present study aimed to examine the mediating role of sleep quality on the relationship between physical activity and SAD among Chinese college freshmen, in order to provide a theoretical basis for emotional management strategies for this population. Our study suggested that the prevalence of SAD among Chinese college freshmen was 33.05%, and female students exhibited a higher prevalence of SAD than their male counterparts, which was consistent with finding from previous study (Li et al., 2022). Physical activity may directly and indirectly influence the risk of developing SAD among college freshmen, with sleep quality acting as a mediating factor. Specifically, mediation analysis suggested that poor sleep quality mediated 24.33% of the association between physical activity and SAD in Chinese college freshmen. The mediation role is categorized as medium-sized according to psychosocial research standards. This finding suggests that improving sleep quality might theoretically mitigate approximately one-quarter of the overall impact of low physical activity on social anxiety. In psychological research examining sleep quality as a mediator, existing studies reported a spectrum of effects ranging from complete mediation to partial mediation (Dang, 2023; Meratwal et al., 2023).

It is well established that physical exercise can be used as a complementary therapy for mental illness (Gordon et al., 2017, 2020; Ramos-Sanchez et al., 2021). A recent cross-sectional study involving first-year college students suggested that higher levels of physical activity were correlated with fewer symptoms of anxiety (Fu et al., 2023). A systematic review performed in 2021 indicated that physical activity may serve as an effective complementary therapy for alleviating symptoms of SAD (Zika and Becker, 2021). Our analysis also demonstrated a negative relationship between levels of physical activity and IAS scores for SAD from two distinct perspectives. Notably, although the difference in mean PARS-3 scores between the SAD and non-SAD groups was not statistically significant, a significant difference was observed in the classification of exercise intensity ratings. These results suggest that the exercise intensity may be an important factor influencing social anxiety among Chinese college freshmen. With regard to the mechanism in question, research has demonstrated that regular physical exercise can influence the acute exercise-induced anti-anxiety effects, specifically in relation to amygdala reactivity (Chen et al., 2019). Furthermore, regular physical exercise can enrich the composition of intestinal flora and promote the abundance of beneficial gut microbiota in humans, resulting in the secretion of various metabolites that play important roles in regulating neural activity, such as serotonin (Schneider et al., 2023). Enhanced serotonin levels are particularly noteworthy, as serotonin is a crucial neurotransmitter involved in mood and emotional regulation (Jenkins et al., 2016), and its balance was disrupted in individuals suffering from anxiety and depression (Baldwin and Rudge, 1995). Our study indicated that ~60% of first-year college students exhibited low levels of physical activity, which may increase their risk of developing social anxiety. Therefore, promoting physical activity among university students may contribute to the prevention and management of social anxiety.

Numerous studies have suggested a bidirectional relationship between physical activity and sleep quality in adults (Maddox et al., 2023). Exercise is known to promote better sleep and enhance sleep quality by regulating circadian rhythms, increasing sleep duration, reducing sleep latency, and promoting more time spent in deep sleep stages. Poor sleep negatively impacted decision-making and overall performance during physical activities by impairing cognitive function. Long-term psychological distress associated with sleep disturbances, particularly insomnia, may instigate or further exacerbate the risk of anxiety, which in turn deteriorates sleep quality (Zhang et al., 2014). Research has demonstrated that sleep plays a critical role in regulating neurotransmitter signaling, primarily by activating the body's stress response. Poor sleep results in impaired emotional regulation (Georgiev et al., 2025). Additionally, sleep deprivation may exacerbate aggressive behavior, which is another manifestation of emotional impairment (Guo, 2024). An investigation about the association between sleep patterns and anxiety levels among college students revealed that individuals with a tendency for delayed sleep onset exhibited high levels of anxiety levels (Silva et al., 2020). The potential mechanism involved may be related to the role of specific brain neurotransmitter, such as the adenosinergic receptor system, in regulating anxiety, arousal, and sleep (Chellappa and Aeschbach, 2022). Regular exercise can enhance mood and reduce anxiety by regulating synaptic plasticity (D'Arcangelo et al., 2017; Bettio et al., 2019) and influencing brain neurotransmitter levels. The notion that sleep quality may act as a mediating factor in the relationship between physical exercise and anxiety highlights the critical roles both play in mental health. Our results also indicated that sleep quality explained 24.33% of the overall role of physical activity on social anxiety among Chinese college freshmen. Incorporating regular exercise not only serves as a direct intervention for reducing social anxiety but also enhances sleep quality, which subsequently promotes sustained emotional and psychological wellbeing among college students.

To our knowledge, this is the first to examine the mediating role of sleep quality on the association between physical activity and SAD among Chinese college freshmen. Furthermore, several potential confounding factors that might affect sleep quality and social anxiety, such as monthly living expenses and involvement in student clubs, were also collected and taken into consideration when examining the association between physical activity and SAD. However, several limitations should be acknowledged when interpreting our findings. First, our study design was a cross-sectional study that was limited to clarify the causal relationship between physical activity and SAD among Chinese college freshmen. Therefore, pertinent longitudinal studies are necessary to elucidate their causal relationship within this population in future research. Second, the results for the PARS-3, IAS and PSQI scores were obtained from self-reported scales, which may be susceptible to recall bias and self-reporting bias. Third, some unmeasured or unknown variables, such as depressive symptoms, and types of exercise with aerobic exercise or anaerobic exercise, may be underestimate or overestimate the mediating role of sleep quality on the association between physical activity and SAD. Finally, considering that the collected questionnaires were obtained from a single university, there may be selection bias, and whether our findings are applicable to freshmen at other universities needs further examination.

## Conclusions

In conclusion, our findings indicate that a lack of physical activity is associated with an increase in social anxiety among Chinese college freshmen, particularly among female students. It is worth mentioning that this association is predominantly mediated by poor sleep quality. Our results highlight the significance of regular exercise and sufficient sleep in regulating mood and alleviating anxiety among this population. Consequently, it is recommended that educational institutions implement programs to promote regular physical activity, specifically encouraging students to engage in either a minimum of 150 min of moderate-intensity aerobic exercise or 75 min of high-intensity aerobic exercise per week (Irwin, 2004; Keating et al., 2005; Barr-Anderson et al., 2018). Additionally, institutions should educate students about the significance of obtaining a minimum of 8 h of sleep to help alleviate social anxiety. Furthermore, fostering supportive environments for exercise and mental health can further enhance the overall wellbeing of this population.

## Data Availability

The raw data supporting the conclusions of this article will be made available by the authors, without undue reservation.
